# Once-daily dolutegravir is superior to once-daily darunavir/ritonavir in treatment-naïve HIV-1-positive individuals: 96 week results from FLAMINGO

**DOI:** 10.7448/IAS.17.4.19490

**Published:** 2014-11-02

**Authors:** Jean-Michel Molina, Bonaventura Clotet, Jan van Lunzen, Adriano Lazzarin, Matthias Cavassini, Keith Henry, Valeriv Kulagin, Naomi Givens, Clare Brennan, Carlos Fernando de Oliveira

**Affiliations:** 1 Service des Maladies, Infectieuses et Tropicales, Hôpital Saint Louis, Paris, France; 2 HIV Unit, Hospital Universitari Germans Trias i Pujol, Irsicaixa Foundation, UAB, UVIC-UCC, Badalona, Catalonia, Spain; 3 Infectious Diseases Unit, University Medical Center Hamburg-Eppendorf, Hamburg, Germany; 4 Department of Infectious Diseases, IRCCS San Raffaele Via Stamira d'Ancona, Milan, Italy; 5 Infectious Disease Service, Lausanne University Hospital, Lausanne, Switzerland; 6 Department of Medicine, Hennepin County Medical Center, Minneapolis, MN, USA; 7 Clinical Center for Prevention and Control of AIDS, Krasnodar, Russian Federation; 8 Clinical Statistics, GlaxoSmithKline, Stockley Park, UK; 9 Infectious Diseases, GlaxoSmithKline, Clinical Development, Research Triangle Park, NC, USA; 10 PPD, Pharmacovigilance, Morrisville, NC, USA

## Abstract

**Introduction:**

Dolutegravir (DTG) 50 mg once daily was superior to darunavir/ritonavir (DRV/r) 800 mg/100 mg once daily through Week 48, with 90% vs. 83% of participants achieving HIV RNA 50 c/mL (*p*=0.025) [1]. We present data through Week 96.

**Material and Methods:**

FLAMINGO is a multicentre, randomized, open-label, Phase IIIb non-inferiority study, in which HIV-1-positive ART-naïve adults with HIV-1 RNA≥1000 c/mL and no evidence of viral resistance were randomized 1:1 to receive DTG or DRV/r, with investigator-selected backbone NRTIs (TDF/FTC or ABC/3TC). Participants were stratified by screening HIV-1 RNA (≤100K c/mL) and NRTI backbone.

**Results:**

A total of 484 adults were randomized and treated; 25% had baseline HIV RNA 100K c/mL. At Week 96, the proportion of participants with HIV RNA 50 c/mL was 80% in the DTG arm vs. 68% in the DRV/r arm (adjusted difference 12.4%; 95% CI 4.7, 20.2%; *p*=0.002). Secondary analyses supported primary results: per-protocol [(DTG 83% vs. DRV/r 70%), 95% CI 12.9 (5.3, 20.6)] and treatment-related discontinuation = failure [(98% vs. 95%), 95% CI 3.2 (−0.3, 6.7)]. Overall virologic non-response (DTG 8%; DRV/r 12%) and non-response due to other reasons (DTG 12%; DRV/r 21%) occurred less frequently on DTG. As at Week 48, the difference between arms was most pronounced in participants with high baseline viral load (82% vs. 52% response through Week 96) and in the TDF/FTC stratum (79% vs. 64%); consistent responses were seen in the ABC/3TC stratum (82% vs. 75%). Six participants (DTG 2, none post-Week 48; DRV/r 4, two post-Week 48) experienced protocol-defined virologic failure (PDVF; confirmed viral load 200 c/mL on or after Week 24); none had treatment-emergent resistance to study drugs. Most frequent drug-related adverse events (AEs) were diarrhoea, nausea and headache, with diarrhoea significantly more common on DRV/r (24%) than DTG (10%). Significantly more participants had Grade 2 fasting LDL toxicities on DRV/r (22%) vs. DTG (7%), *p*<0.001; mean changes in creatinine for DTG (~0.18 mg/dL) observed at Week 2 were stable through Week 96.

**Conclusions:**

Once-daily DTG was superior to once-daily DRV/r in treatment-naïve HIV-1-positive individuals, with no evidence of emergent resistance to DTG in virologic failure and relatively similar safety profiles for DTG and DRV/r through 96 Weeks.
